# Comparing postoperative outcomes of two fully hydroxyapatite-coated collarless stems in total hip arthroplasty through propensity score matching analysis with 2 years follow-up

**DOI:** 10.1038/s41598-022-24569-9

**Published:** 2022-11-21

**Authors:** Takashi Imagama, Yuta Matsuki, Takehiro Kaneoka, Takehiro Kawakami, Kazushige Seki, Toshihiro Seki, Kenji Hirata, Tomoya Okazaki, Hiroshi Tanaka, Takashi Sakai

**Affiliations:** 1 Department of Orthopaedic Surgery, Yamaguchi University Graduate School of Medicine, 1-1-1, Minamikogushi, Ube, 7558505 Japan; 2 Department of Orthopaedic Surgery, Yamaguchi Prefectural Grand Medical Center, Hofu, Japan

**Keywords:** Medical research, Outcomes research

## Abstract

A fully hydroxyapatite (HA)-coated stem such as Corail stem, that compacts the cancellous bone around the stem in total hip arthroplasty (THA), is reported to have good long-term results for more than 20 years. Although various fully HA-coated stems have being used recently, it is unclear whether there are differences in the postoperative outcomes. In this study, 224 patients (234 hips) with THA using either the Corail collarless stem or the Hydra stem were enrolled. And then we performed a retrospective comparison of the data at 2 years postoperatively using propensity score matching analysis. The postoperative modified Harris hip scores in 84 hips each group were 93.6 ± 8.2 points in the Corail group and 92.8 ± 10.1 points in the Hydra group, and there was no significant difference between the two groups. However, there was significantly less stem subsidence and rate of 3rd degree or greater stress shielding in the Corail group. Although these two stems were similar collarless fully HA-coated stems and clinical outcomes were favorable results in both groups at 2 years postoperatively, radiographic evaluations showed statistically significant differences between the two groups.

## Introduction

Total hip arthroplasty (THA) is primarily performed on osteoarthritis patients with intense pain and marked functional impairment of the hip, and many studies reported good long-term postoperative clinical outcomes. It is one of the most successful surgeries of the twentieth century^1^.

There is a great deal of variation on the stem used in THA, and different components are used by various institutions. In 2012, cementless stems were classified by Khanuja et al. as Type 1: single wedge, Type 2: double wedge metaphyseal filling, Type 3A: tapered round, Type 3B: tapered spline/cone, Type 3C: tapered rectangle, Type 4: cylindrical fully coated, Type 5: modular, and Type 6: anatomic^2^. However, a fully hydroxyapatite (HA)-coated stem such as Corail stem (DePuy Synthes, IN, USA), does not correspond to this classification and can be designated as a special-concept stem. Corail stem is a cementless stem that was available in the launching since 1986 and is currently the most-used implant among cementless stems according to data from the United Kingdom and Australian registries^3,4^. The Corail stem is double taper shaped, which is designed with the proximal lateral sides of the stem cut at a slant. And it is chiefly characterized by a rasp that compacts the cancellous bone of the femur, thereby forming a compressed layer of cancellous bone called cancellous bone bed between the stem and the cortical bone. It is described that the load applied to the stem is uniformly transmitted to the femur, and the stress applied to the cortical bone is disseminated^5^. In addition, the circumferential HA coating reduces the formation of fibrous tissue in the bone and implant surfaces and promotes bone regeneration on the HA surface^6^. According to a report by Vidalain JP, the Corail stem had a survival rate of 96.5% at 23 years postoperatively and reported less stress shielding, cortical hypertrophy, and thigh pain, indicating good long-term outcomes^7^.

Meanwhile, other fully HA-coated stems similar to Corail stem have come into use since the year 2000. Of these, the Hydra stem (Adler, Milan, ITA) has a similar design as Corail stem, with a double taper and cutting on the proximal lateral stem and uses compaction broaching. However, it differs in having a changeable neck and a thinner HA layer compared to Corail stem, as well as having a sharp edge for the compaction broach. It is still unclear how these differences between Corail and Hydra stems affect the postoperative outcomes.

Corail stem has both collared and collarless stems. Although the collared type is reported to have a better initial fixation performance in mechanical testing using cadavers^8^, both have been clinically reported to demonstrate good postoperative outcomes and there is no consensus on whether there is a significant difference^7^. Conversely, when removing the stem in revision THA, the collarless stem is superior in that the difficulty is lower.

Therefore, this study aimed to compare the outcomes of the Corail collarless stem and the Hydra stem retrospectively with propensity score matching analysis in patients who underwent THA and elucidate whether there are differences in the 2-year postoperative clinical and radiographic outcomes and complication rates.

## Results

### Patient demographics

The propensity score matching results enrolled 80 patients (84 hips) in the Corail group and 83 patients (84 hips) in the Hydra group. The mean age was 66.5 ± 10.1 years in the Corail group, and 65.7 ± 10.6 years in the Hydra group (p = 0.668). There were 5 males and 75 females in the Corail group, whereas there were 5 males and 78 females in the Hydra group (p > 0.999). The mean body mass index (BMI) was 23.6 ± 3.8 kg/m^2^ in the Corail group and 23.3 ± 3.5 kg/m^2^ in the Hydra group (p = 0.665). The diagnosis were osteoarthritis of the hip in 74 and 78 hips and osteonecrosis of the femoral head in 10 and 6 hips in the Corail and Hydra groups, respectively, with no statistically significant difference between the two groups (p = 0.431). According to the Dorr classification, types A, B, and C were observed in 20, 58, and 6 hips, respectively, in the Corail group, and 17, 64, and 3 hips, respectively, in the Hydra group, with no significant difference between the two groups (p = 0.463). The mean preoperative modified Harris hip score (mHHS) was 46.9 ± 12.8 points in the Corail group and 44.2 ± 13.2 points in the Hydra group, with no differences between the two groups (p = 0.419). The mean follow-up period was 2.4 ± 0.3 years for the Corail group and 2.3 ± 0.2 years for the Hydra group (p = 0.276) (Table 1).

**Table 1:** Baseline demographics of patients after propensity score matching.

	Corail group	Hydra group	P value
Number of patients/hips	80/84	83/84	–
Age (y)	66.5 ± 10.1	65.7 ± 10.6	0.668
Male:female	5:75	5:78	> 0.999
Body mass index (kg/m^2^)	23.6 ± 3.8	23.3 ± 3.5	0.665
Diagnosis (hips)	OA: 74, ONFH: 10	OA: 78, ONFH: 6	0.431
Dorr classification (hips)	A: 20, B: 58, C: 6	A: 17, B: 64, C: 3	0.463
Surgical approach	DAA	DAA	–
Pre mHHS	46.9 ± 12.8	44.2 ± 13.2	0.419
Follow-up period (y)	2.4 ± 0.3	2.3 ± 0.2	0.276

*OA* osteoarthritis, *ONFH* osteonecrosis of the femoral head, *DAA* direct anterior approach, *mHHS* modified Harris hip score.

### Outcomes

The mean mHHS at 2 years postoperatively was 93.6 ± 8.2 points for the Corail group and 92.8 ± 10.1 points for the Hydra group. No significant difference was observed in both groups (p = 0.744). In radiographic analyses, there were no cases of stem loosening in both groups. Excluding cases with intraoperative fractures, a mean subsidence of 0.8 ± 0.7 mm in the Corail group and 1.9 ± 2.1 mm in the Hydra group, it was significantly lower in the Corail group (p = 0.001). Whereas radiolucent lines appeared in zone 1 only in 7 (8.3%) and 14 hips (16.7%) in the Corail and Hydra groups, respectively, and 1 hip (1.2%) each in both groups in zones 1 and 7, with no significant difference between the groups (p = 0.116). Regarding stress shielding, in the Corail group 3rd degree was 1 hip (1.2%) and in the Hydra group, 3rd and 4th degrees were 11 hips (13.1%) and 1 hip (1.2%) respectively. The rate of stress shielding was significantly lower in the Corail group (p = 0.002). No cortical hypertrophy was observed in both groups. Varus stem alignment were observed in 1 hip (1.2%) in the Corail group, and 5 hips (6.0%) in the Hydra group, with no significant differences between the two groups (p = 0.109). No valgus stem alignment was observed in both groups (Table 2). The statistical power of mHHS, stress shielding, and subsidence using G*power 3.1.9.7 (Düsseldorf, Germany) was 0.78, 0.93, and 0.88, respectively.

**Table 2:** Postoperative outcomes.

	Corail group	Hydra group	P value
**Postoperative mHHS**	93.6 ± 8.2	92.8 ± 10.1	0.744
**Stem loosening (hips)**	0	0	–
**Subsidence (mm)**	0.8 ± 0.7	1.9 ± 2.1	0.001
**Radiolucent line (hips)**	8	15	0.116
Zone 1/2/3/4/5/6/7	8/0/0/0/0/0/1	15/0/0/0/0/0/1	
**Stress shielding (hips)**			
0°/I°/II°/III°/IV°	10/28/32/1/0	2/16/41/11/1	
0–II°	70	59	
≥ III°	1	12	0.002
**Cortical hypertrophy**	0	0	–
**Stem alignment (hips)**			
Varus	1	5	0.109
Valgus	0	0	–

*mHHS* modified Harris hip score.

### Complications

Superficial surgical site infection (SSI) was observed in 1 hip in the Corail group, and no postoperative dislocations were observed in both groups. An intraoperative periprosthetic femoral fracture (PPF) was observed in 2 hips in the Corail group and 5 hips in the Hydra group. Thigh pain was observed in 2 hips in only Hydra group. No significant difference was observed between the groups for all complications (SSI: p = 0.319, intraoperative PPF: p = 0.267, thigh pain: p = 0.160) (Table 3).

**Table 3:** Complications.

	Corail group	Hydra group	P value
Surgical site infection	1 (superficial)	0	0.319
Dislocation	0	0	–
Intraoperative PPF	2	5	0.267
Thigh pain	0	2	0.160

*PPF* periprosthetic femoral fracture.

## Discussion

We performed propensity score matching analysis and compared the 2-year postoperative outcomes of the fully HA-coated Corail collarless stem and Hydra stem used in patients who underwent primary THA. Although no significant differences were observed in the 2-year postoperative mHHS between the two groups, the incidence of subsidence and stress shielding was statistically and significantly lesser in the Corail group than in the Hydra group. This is the first report on the differences in short-term postoperative outcomes of the Corail stem and the Hydra stem.

Regarding the long-term outcomes of the Corail stem, Vidalain JP reported an average mHHS of 41.3 points preoperatively and 85.1 points at 24 years postoperatively^7^, meanwhile Froimson et al. reported that the d’Aubergine and Postel scores had significantly improved from an average of 5.5 points preoperatively to 16.5 points at 10 years postoperatively^9^. Both studies demonstrated good long-term postoperative outcomes. In the present study, the Hydra group has also exhibited good short-term postoperative clinical outcomes similar to the Corail group.

In radiographic analyses, favorable results were achieved with no stem loosening observed in both groups. Even in terms of long-term postoperative outcomes, a stem survival rate of 96.4% at 23 years postoperatively has been reported for the Corail stem^7^, whereas good outcomes have also been reported for THA using the Hydra stem and Fixa Ti-POR cup, with a 10-year postoperative survival rate of 95.6%^10^. However, the stress shielding of 0 to 2nd degree was 98.6% with the Corail stem, it was 83.1% with the Hydra stem in this study, and 3rd degree or greater stress shielding was observed more with the Hydra stem than the Corail stem. And then, radiolucent lines in both groups were mostly observed in zone 1. Although no significant difference was observed, the positive rate tended to be lower in the Corail group at 8.3% versus 16.7% in the Hydra group. These results may be due to the difference in the HA thickness. HA is a biocompatible and bioactive material, and its chemical crystal composition is largely similar to the mineral composition of bone, with strong osteoconduction^11^. Thus, it promotes bone ongrowth on the HA stem coating through the proliferation of osteoblasts and bone regeneration. The HA coating on the stem must be of a certain thickness because it will partially dissolve with time. The Corail stem has a HA thickness of 155 µm, the thickest among similar fully HA-coated stems. Frayssinet et al. reported that sufficient HA coating remained even at 2 years postoperatively after Corail stem implantation^12^. However, the Hydra stem has a HA thickness of 80 µm, which is thinner than the Corail stem. It is entirely possible that this affected the results of radiolucent line in this study.

**Figure 1:**
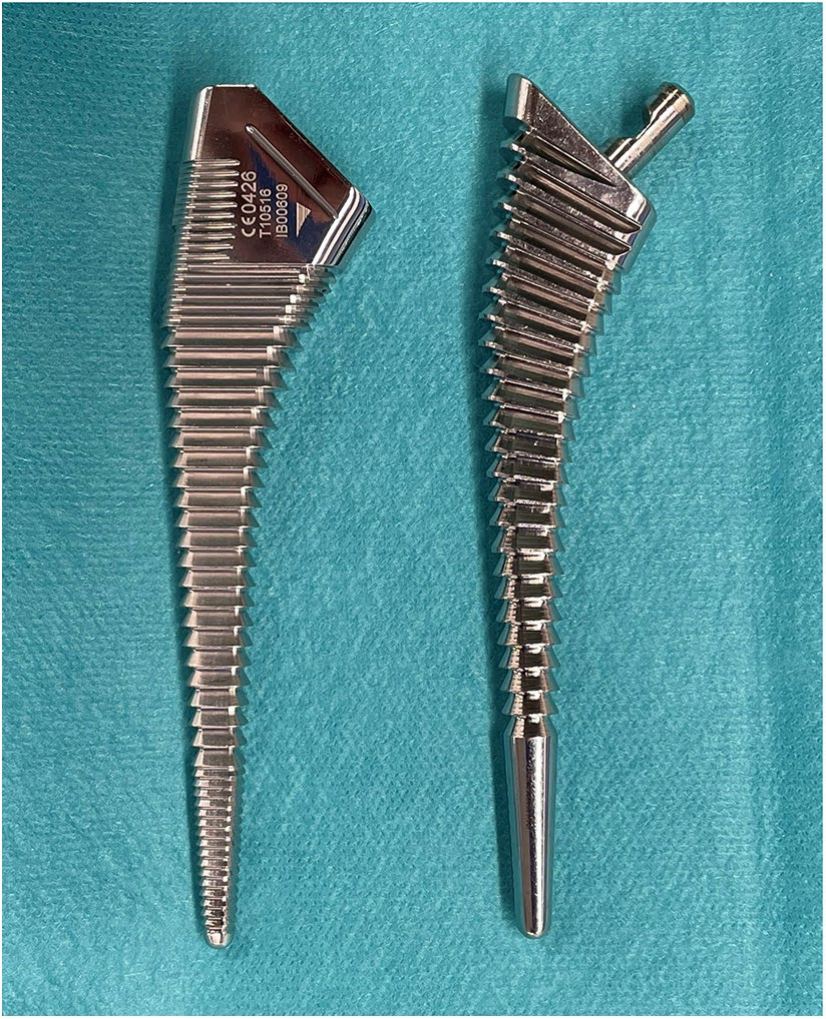
Antero-posterior view of each rasping broach. The broach on the left is Hydra stem and the one on the right is Corail stem. Proximal part of Hydra broach has almost no edges unlike Corail broach.

**Figure 2:**
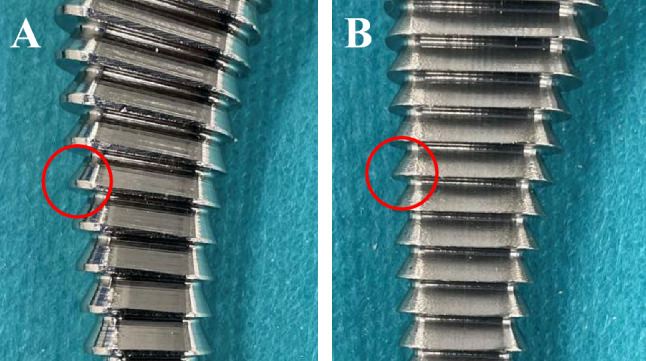
Different shapes of the edge at each broach. The edge is relatively dull in the Corail broach (**A**), whereas it is sharp in the Hydra broach (**B**).

 The present study showed that the subsidence was significantly lesser with the Corail stem. Because of the initial fixation mechanism of fully HA-coated stems in which compaction broaching depends on the cancellous bone bed, it is important to compress the cancellous bone to the maximum extent. A study of inserting implants in the femoral condyle of dogs reported better early bone-implant contact and increased implant fixation in the group which had bone compaction compared to only drilling^13^. Although compaction broaching was performed using both stems, the Hydra stem has few edges on the most proximal part of the compaction broach unlike the Corail stem (Fig. 1). This may mean that Hydra stem has less compaction of the cancellous bone at the proximal part. Moreover, the peripheral edge on the Corail broach is comparative dull, while it is sharp in the Hydra stem (Fig. 2). Hence, the Hydra stem may scrape the cancellous bone off, unlike the Corail stem. These differences likely affected the quality of cancellous bone bed and affected initial fixation of the stem.

**Figure 3:**
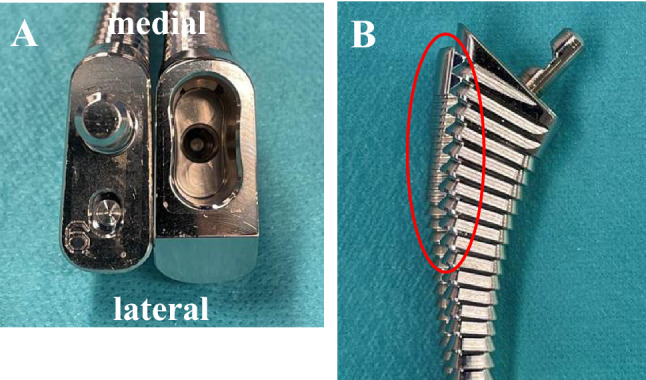
Difference in size of each same number broach. Overlapping the same number 9 broaches with the Corail on top, Hydra broach is more lateral overhanging in proximal part (red oval) than Corail broach in the antero-posterior view (**A**). In the view of each broach from cranial, the antero-posterior width is larger in the hydra broach (left) than the Corail broach (right) (**B**).

In addition, although there was no statistically significant difference in the rates of both varus alignment and intraoperative PPF, they tended to be higher in the Hydra group. These could be influenced by the shape of implants. First, it would be considered that the antero-posterior width of proximal part is greater in Hydra stem than Corail stem, comparing the broaches of same number (Fig. 3A). This may increase the risk that the stem contacts with the proximal medial femoral cortical bone, especially in smaller patients. Consequently, Hydra stem may apply stress to the medial cortical bone of the femur during stem insertion and is also a possible reason why fractures tended to be slightly more common in the Hydra group. Second, the shape of Hydra stem extended farther out on the proximal lateral side compared to the Corail stem (Fig. 3B). A study focused on the shape of the rasping broach of fully HA-coated stems, including the Corail stem of a similar shape used in THA where DAA was performed. It reported that there were more varus insertions with stems which the proximal lateral side extension of the broach was greater^14^. The difference of the shape between Hydra and Corail stems was particularly pronounced in small size stems such as sizes 8 and 9. In fact, among the 10 hips with varus insertion with the Hydra stem in this study, 4 hips were treated using size 8, 3 hips with size 9, and 1 hip each with sizes 11, 12, and 14. In addition, 5 hips of intraoperative fracture with Hydra stem were used with sizes 8 in 3 hips, size 9 in 1 hip, and size 14 in 1 hip. These considerations are speculative and further studies are needed, as frequencies were not significantly different in the two groups. However, we consider that it is important to become familiar with characteristics of each stem shape in order to correctly insert the stem. Magill et al. reported the stem alignment and postoperative outcomes with the Corail stem and stated that in 4802 hip arthroplasties, there were 15 hips in with aseptic loosening of which 9 hips had varus insertion. They stated that varus insertion was a risk factor for stem loosening^15^. Although no loosening in both groups was showed in the present study, it was only for short follow-up period. Long-term careful follow-up is needed for varus insertion cases in the future.

In the present study, the incidence of thigh pain was low in both groups with no significant difference. Thigh pain is a known postoperative complication of cementless stems and is a major factor associated with postoperative satisfaction. Although the cause is considered multifactorial, the concentration of load transmission, primarily on the distal part of the stem, has been reported as one of the causes^16^. Won et al., compared a proximal coated, tapered, cementless short stem with a standard stem and reported the incidences of thigh pain to be 16% and 14%, respectively^17^. On the other hand, Vidalain JP reported that thigh pain was observed in only 2 out of 127 hips at 20 years postoperatively using the Corail stem, and the rate was similar to the results of the present study. In addition, although cortical hypertrophy, where the cortical bone becomes thicker when stress is concentrated at the distal part of the stem has been reported to correlate to thigh pain in THA using short tapered-wedge stem^18^, no cortical hypertrophy was observed with either Corail or Hydra stem in this study. These results may be considered to be in line with the concept of the Corail type fully HA-coated stems, where the load is distributed on the entire body of the stem.

 This study has several limitations. First, it was a retrospective study with small sample size. However, propensity score matching was performed, and it is considered that there were no significant intergroup differences in baseline demographics, with slight variations among the patients in each group. In addition, when the necessary sample size was calculated with an effect size of 0.5 and power of 0.8 using G*Power, the total sample size required was 134 cases, and therefore, our study can be considered a significant investigation. Second, the follow-up period was short. The significant differences in the current two groups were observed in subsidence, stem insertion angle, and stress shielding. It is known that Corail subsidence appears early, within 3 months postoperatively, and does not progress thereafter^19^, and the stem insertion angle is affected by the intraoperative insertion angle. Therefore, evaluating these factors at 2 years postoperatively was believed to be valid. However, since stress shielding may change over the years, long-term follow-up is necessary going forward. Finally, the retrospective study and the use of stem based on criterion of surgeon alone may introduce selection bias. However, on evaluation of patient and surgical factors that have been previously demonstrated to postoperative outcomes in THA, the groups were statistically similar after propensity score matching. Therefore, we consider that the selection bias is unlikely to be clinically significant.

In conclusion, we performed propensity score matching and compared the 2-year postoperative outcomes of the Corail collarless stem and Hydra stem, which are fully HA-coated stems, in patients with THA. Both groups demonstrated good clinical outcomes and low rates of complications, with no significant difference. However, there was lesser stem subsidence and lower incidence of stress shielding of 3rd degree or greater in the Corail group as observed in the radiographic examination. It is necessary to know the possibility of differences in postoperative results due to differences in the shape of the rasping broach or the other factor, even in fully HA-coated stems, which is a similar stem fixation concept.

## Methods

### Patient selection criteria

**Figure 4:**
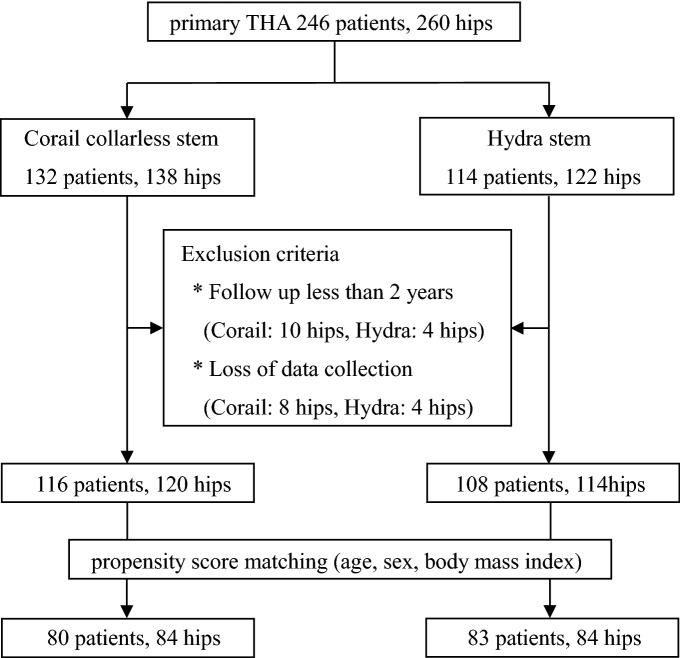
Flowchart showing the inclusion and exclusion of patients with follow up.

We performed a retrospective investigation on 246 patients (260 hips) who underwent primary THA through DAA with either a Corail collarless stem or a Hydra stem performed at our hospital as well as the Yamaguchi Prefectural Grand Medical Center between July 2014 and October 2019. The Corail stem was used in 132 patients (138 hips) and the Hydra stem in 114 patients (122 hips). The exclusion criteria were cases where the follow-up period was less than 2 years and cases whose data was lost. Finally, 224 patients (234 hips) were included in the study (Corail collarless stem: 116 patients [120 hips]; Hydra stem: 108 patients [114 hips]). A total of 16 patients (18 hips) in the Corail group and 6 patients (8 hips) in the Hydra group were excluded from the analysis. Among the excluded patients, 8 patients (10 hips) in the Corail group and 3 patients (4 hips) in the Hydra group were followed up within 2 years, and 8 patients (8 hips) and 3 patients (4 hips) respectively were missing data on the analysis. The included patients were divided into two groups, Corail and Hydra, after performing propensity score matching by age, sex, and BMI using JMP 13.0 data analysis software (SAS institute, NC, USA) (Fig. 4). This study was approved by the Yamaguchi university graduate school of medicine institutional review board (H2020-068).

### Surgical technique

All patients underwent THA with DAA. The patient was placed in a supine position on a normal operation table. In the Corail group, all patients were fitted with a collarless stem and PINNACLE cup (DePuy Synthes, IN, USA), and those in the Hydra were fitted with a collarless changeable neck stem and FIXA Ti-Por cup (Adler, Milan, ITA). The bearing surfaces in the Corail group used the BIOLOX delta ceramic head and a polyethylene liner, and in the Hydra group used BIOLOX delta ceramic head and insert. All surgeries were performed by three surgeons with at least 17 years of experience in hip arthroplasty. Each surgeon performed preoperative planning with three-dimensional templating software ZedHip (LEXI, Tokyo, Japan), and they selected one of the fully HA coated stems with better adaptation. If both stems were not adaptive, other stem was used.

### Evaluation

The following parameters were evaluated: age at the time of surgery, sex, BMI, diagnosis, preoperative and 2-year postoperative mHHS. In addition, the radiological parameters such as the Dorr classification for the medullary cavity morphology of the femur^20^ as well as the presence of cortical hypertrophy, stress shielding, subsidence, and radiolucent lines. Radiolucent line was defined as 2 mm or greater on anteroposterior radiographs of the hip at 2 years postoperatively using the Gruen classification^21^. The presence of stem loosening and the varus–valgus angles of the stem (3° or greater was significant) were also evaluated. The stress shielding with the Engh classification^22^. Radiological evaluations were performed by two orthopaedic surgeons, and in the event of a difference of opinion, a conclusion was reached by discussion. Complications included SSI, dislocation, intraoperative PPF, and thigh pain. These evaluations were elicited during clinical appointments.

### Statistical analysis

Continuous variables are expressed as means ± standard deviation. The Student’s *t* test was used to compare continuous variables between the two groups, and the Chi-square test was used to compare the incidence rates of the each parameter. A p-value of ≤ 0.05 was considered as statistically significant. All analyses were performed using Graphpad prism Ver.8 (GraphPad, CA, USA) and JMP 13.0.

### Ethics approval

This study was performed in line with the principles of the Declaration of Helsinki. Approval was granted by the Ethics Committee and Institutional Review Board of Yamaguchi University (H2020-068).

### Informed consent

Informed consent was obtained from all individual participants included in the study.

## Data Availability

The datasets generated and analysed during the current study are not publicly available, because this study contains important patient’s personal information, which could lead to identification of patients. The informed consent has not been also obtained to disclose detailed patient information to the public. However, they are available from the corresponding author on reasonable request.
